# A New Approach to Define and Diagnose Cardiometabolic Disorder in Children

**DOI:** 10.1155/2015/539835

**Published:** 2015-04-06

**Authors:** Lars Bo Andersen, Jeppe Bo Lauersen, Jan Christian Brønd, Sigmund Alfred Anderssen, Luis B. Sardinha, Jostein Steene-Johannessen, Robert G. McMurray, Mauro V. G. Barros, Susi Kriemler, Niels Christian Møller, Anna Bugge, Peter Lund Kristensen, Mathias Ried-Larsen, Anders Grøntved, Ulf Ekelund

**Affiliations:** ^1^Center of Research in Childhood Health, Department of Sport Sciences and Clinical Biomechanics, University of Southern Denmark, Campusvej 55, 5230 Odense M, Denmark; ^2^Department of Sports Medicine, Norwegian School of Sport Sciences, Sognsveien 220, 0806 Oslo, Norway; ^3^Exercise and Health Laboratory, CIPER, Fac Motricidade Humana, Universidade de Lisboa, Estrada Dacosth, Cruz-Quebrada, 1499 Lisbon, Portugal; ^4^Department of Exercise and Sport Science, University of North Carolina, 025 Fetzer Gym, CB No. 8700, Chapel Hill, NC 27599-8700, USA; ^5^School of Physical Education, University of Pernambuco, Campus Universitario HUOC-ESEF, Arnobio Marques 310, Santo Amaro, 50.100-130 Recife, PE, Brazil; ^6^Epidemiology, Biostatistics and Prevention Institute, University of Zürich, Hirschengraben 84, 8001 Zürich, Switzerland; ^7^The Centre of Inflammation and Metabolism and Trygfondens Center for Aktiv Sundhed, Department of Infectious Diseases and CMRC, Rigshospitalet, Faculty of Health Sciences, University of Copenhagen, Tagensvej 20, 2100 Copenhagen, Denmark; ^8^MRC Epidemiology Unit, Institute of Metabolic Science, Addenbrooke's Hospital, University of Cambridge, Hills Road, Cambridge CB2 0QQ, UK

## Abstract

The aim of the study was to test the performance of a new definition of metabolic syndrome (MetS), which better describes metabolic dysfunction in children. *Methods*. 15,794 youths aged 6–18 years participated. Mean *z*-score for CVD risk factors was calculated. Sensitivity analyses were performed to evaluate which parameters best described the metabolic dysfunction by analysing the score against independent variables not included in the score. *Results*. More youth had clustering of CVD risk factors (>6.2%) compared to the number selected by existing MetS definitions (International Diabetes Federation (IDF) < 1%). Waist circumference and BMI were interchangeable, but using insulin resistance homeostasis model assessment (HOMA) instead of fasting glucose increased the score. The continuous MetS score was increased when cardiorespiratory fitness (CRF) and leptin were included. A mean *z*-score of 0.40–0.85 indicated borderline and above 0.85 indicated clustering of risk factors. A noninvasive risk score based on adiposity and CRF showed sensitivity and specificity of 0.85 and an area under the curve of 0.92 against IDF definition of MetS. *Conclusions*. Diagnosis for MetS in youth can be improved by using continuous variables for risk factors and by including CRF and leptin.

## 1. Introduction

Metabolic syndrome (MetS) was first described by Reaven in the mid 1980s [1]. MetS is a conceptual framework, which links several apparently unrelated biological events into a single pathophysiological assemble; that is, several cardiovascular disease (CVD) risk factors seemed to be increased simultaneously in some individuals. Despite different definitions most agree on the individual components constituting the MetS, which include dyslipidemia (triglycerides and cholesterol), hypertension, glucose intolerance, and adiposity [2–5]. Originally, the different CVD risk factors were mainly treated with drugs and it therefore seemed logical to use the cutoff point for each risk factor in the definition of the criteria for MetS. Criteria have been suggested by several organizations and researchers and have changed over time even if they all build on the same concept. Insulin resistance triggered a common mechanism affecting blood pressure (BP), high density lipoprotein cholesterol (HDL), triglycerides (TG), and glucose tolerance, and central obesity may cause the insulin resistance and therefore be a central part of MetS. This concept subsequently evolved to encompass a number of multiple definitions [2] established by the WHO [3, 5], the National Cholesterol Education Program Adult Treatment Panel III (ATP III) [4], and the International Diabetes Federation (IDF) [5]. Modified criteria based on the same concept as in adults have been suggested in children [6–9].

Existing MetS definitions have shortcomings, especially for children, and because studies use different definitions comparison between studies is difficult. All definitions are based on dichotomisation of the CVD risk factors and to be clinically diagnosed with the MetS the thresholds for at least three risk factors including obesity must be attained. Limitations include (1) reduction of available information of risk by dichotomizing variables; (2) different risk factors that are given different weight (i.e., prevalence of the risk factors differs, which means that few are selected based on the rare risk factors); (3) thresholds for the individual risk factors that are arbitrarily chosen in children, where no hard endpoints exist; (4) selection of risk factors that exclude potentially important variables; for example, the use of fasting glucose in children rather than fasting insulin or HOMA score as measure of impaired glucose regulation may conceal important information; many children with severe insulin resistance are still able to regulate their fasting blood glucose well [10]; (5) different definitions that use different blood variables and fatness variables. This problem makes it difficult to compare prevalence between populations. These shortcomings result in substantial misclassification, and comparison between studies using different criteria is difficult. When children are analysed, there is also a major difference between the number of children diagnosed with MetS compared to the number where a clustering of risk factors occurs [11, 12].

The current definitions in use preclude accurate estimates of the MetS prevalence between populations and the dichotomisation of variables may attenuate or preclude real associations between lifestyle behaviours and metabolic risk.

The aim of this study was to develop a novel method for identifying young people with increased metabolic risk and solve weaknesses of former definitions. Furthermore, we tested a simple noninvasive screening tool to identify children where further investigation is indicated. The steps in the analysis were (a) to calculate the number of children where risk factors were not independently distributed; (b) to construct age adjusted* z*-scores for each risk factor based on common means and SDs for the whole database for genders separately; (c) to define a cutoff point in mean of summed* z*-scores, which selected the same number of children as calculated above, where risk factors were not independently distributed; (d) to construct a program where absolute values of the risk factors can be entered, and the program calculates the mean* z*-score of the included variables (age and sex adjusted) to evaluate if the child has a disorder; (e) to do sensitivity analyses to evaluate if other risk factors may improve diagnostic criteria; and (f) if it matters to substitute a measure of a certain trait with another such as including* z-*score of body mass index (BMI) instead of waist circumference. The program makes it easy for the general practitioner to use the diagnostic tool. Last, we tested a noninvasive measure of metabolic risk based on physical fitness and fatness.

This approach will make it possible to use the available information and get a better measure of the risk of the child. Information is not reduced when we use continuous variables and the composite* z-*score can be used as a continuous or dichotomous variable. It is hypothesized that the* z-*score of the different variables covering the same trait will only differ slightly; that is, the mean* z-*score only changes marginally if BMI is entered instead of waist, which makes the program flexible and enables comparison between studies using different measures. Further, this approach makes it possible to include other risk factors in the calculation of mean* z*-score or exclude them if data is not available. The constructed program is available on the Internet or as an APP for smartphone, and the general practitioner can enter the available information, which should include as many of the risk factors as possible.

## 2. Methods

### 2.1. Participants

We pooled cross-sectional data from 23 population based cohorts in children and adolescents aged 6–18 years. The 23 cohorts were merged into 18 cohorts before analysis. Ten cohorts (*N* = 4806) of 9- and 15-year-old boys and girls from the European Youth Heart Study (EYHS) (1997 through 2007) were included. Nine-year-olds and 15-year-olds, respectively, assessed at two different time points from the same country were pooled before analysis. Additionally, four cohorts of 9- and 15-year-olds from EYHS Norway (*N* = 3020) were included but analysed as four separate cohorts, as two cohorts lacked important blood variables. The Copenhagen School Child Intervention Study (CoSCIS) (*N* = 1816) included three measurements collected in 2001, 2004, and 2008. Data from the US NHANES (*N* = 4801) collected in 2003 and 2005 were merged and recoded into three age groups (<10 years, 10–14 years, and >14 years). Finally, children from Switzerland (KISS) were sampled from 1st (mean age 8 years) and 5th grade school children (mean age 12 years) and were measured three times over two years (*N* = 1351). Successive measurements of a child were regarded as multiple individuals. In total 7902 girls and 7892 boys were included in the analysis. Data collection procedures and analytical methods have previously been described in detail (EYHS [13], CoSCIS [14, 15], KISS [16], NHANES [17], Norwegian EYHS 2000 [18], and Norwegian EYHS 2005 [19]). Ethical approval has been obtained for all included cohort studies in their respective countries.

### 2.2. Analytical Process and Statistics

Statistical analysis was performed in IBM SPSS Statistics 22 and Stata 12 (meta-analysis).

Common estimates were calculated using random effect meta-analysis [13].

Analysis was progressed by the following steps.

1. First step is calculation of the number of children where risk factors were not independently distributed; that is, risk factors clustered. MetS risk factors follow a binomial distribution if they are independently distributed. If the observed number of children with a given number of risk factors exceeds the expected, risk factors exhibit clustering. For this analysis we defined the upper quartile of waist circumference, systolic blood pressure (BP), triglyceride (TG), and HOMA together with the lower quartile of high density lipoprotein cholesterol (HDL) to be at risk for each cohort separately. Proportion of expected subjects with a specific number of risk factors according to the binomial distribution was (*n*!∗*p* ^*r*^∗(1 − *p*)^*n*−*r*^)/(*r*!∗(*n* − *r*)!), where *n* is the possible number of risk factors (5), *p* is the proportion having the risk factor (25%), and *r* is the number of risk factors the probability is calculated for. The expected proportions of children having 0 to 5 risk factors were 0.178, 0.356, 0.297, 0.132, 0.057, 0.0044, and 0.0002, respectively. We then divided the observed number of children with a specific number of risk factors with the expected and calculated 95% confidence intervals as exp(Ln(OR)+/−1.95∗SE(Ln(OR))), where SE(Ln(OR) = SQRT(1/*n*_*i*_) + (1/(*N* − *n*_*i*_))). *N* is the total number of children in the cohort and *n*_*i*_ is the number of children with the specific number of risk factors.
2. The association between age and each risk factor was calculated using linear regression in order to compute age adjusted risk factor levels for each child. All risk factors were adjusted to the age of 12 years for each gender to enable comparison of levels between cohorts of different age by the following formula: risk factor_adjusted_ = risk  factor − (*β*∗(age − 12)). BMI, waist circumference, sum of 4 skinfolds, TG, glucose, HOMA, C-reactive protein (CRP), APOA1, APOB, and leptin were skewed and therefore log-transformed before* z*-scores were constructed.* z*-scores were constructed using mean and SD of the age adjusted risk factors for the total sample. The mean of the 5 risk factors included in the IDF definition of MetS [6] was calculated using HOMA rather than fasting glucose as an indicator of glucose metabolism.
3. Third step is definition of a cut-point in mean of summed* z*-scores, which selected the number of children with clustered CVD risk as calculated in step (1).
4. Fourth step is determination of the significance of substituting a measure of a certain trait with another, for example, including* z*-score of body mass index (BMI) or skinfold instead of waist circumference or HOMA instead of fasting glucose.
5. Fifth step is sensitivity analyses to evaluate if addition of other risk factors improved diagnostic criteria. For this purpose we examined the associations between CRF (independent variable) by logistic regression and calculated odds ratio (OR) for the three lower quartiles of CRF against the MetS variables (dependent variable). Additional risk factors (APOA1, APOB, leptin, and CRP) beyond those included in the IDF criteria were tested to examine if their inclusion strengthened the magnitude of association between CRF and the mean* z*-score of the traditional MetS risk factors. In all analyses a threshold in the mean* z*-score, identifying the same proportion of children, was identified. Inclusion of CRF in the mean* z*-score was tested by removing waist circumference from the MetS and using it as independent variable to see if the association became stronger when fitness was included in the MetS outcome.
6. Prognostic value of a MetS outcome including only noninvasive variables (waist/height and inverse CRF) was also tested. We used waist circumference/height as a proxy for fatness, because this variable is independent of age [20]. The mean* z*-score of ((waist/height) + (1/CRF))/2 was examined in a receiver operating characteristic (ROC) analysis against the IDF definition of the MetS and the clustered MetS score variable constructed from* z-*scores of log TG, log waist, systolic BP, log HOMA, inverse HDL, and inverse fitness.

All analytical steps were built into an Internet-based program which calculates MetS score in order to facilitate the use in general practice (http://www.video4coach.com/zscore/index.html).

The proportion of children categorised as having the metabolic syndrome (IDF definition) was calculated according to Zimmet et al. [6], where age- and sex-specific 90th percentiles for waist circumference were defined according to Fernández et al. [21].

## 3. Results

Age, anthropometrics, and CVD risk factors are described for each cohort in web-appendix (see web-only Table A in Supplementary Material available online at http://dx.doi.org/10.1155/2015/539835). Characteristics of CVD risk factors are also described after adjustment for age to enable comparison of levels between cohorts of different age (Table 1). The association between age and each risk factor is shown in Table 2.

**Figure 1:**
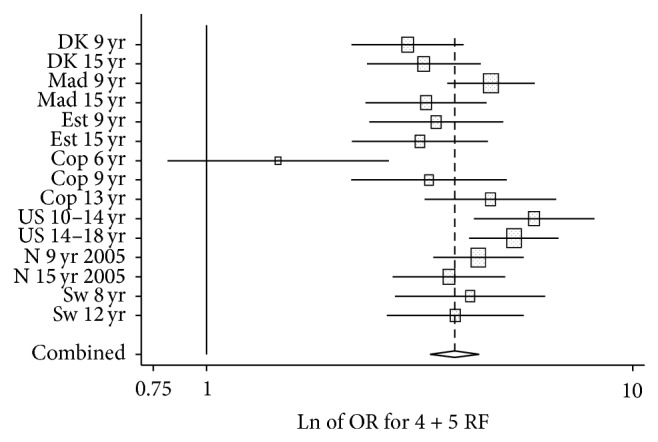
Odds ratios of observed number of children with 4 or 5 risk factors divided with expected number for each cohort.

The number of children according to number of risk factors defined as the upper quartile (0–5 risk factors), the corresponding mean* z*-score, and the proportion defined as having MetS according to IDF definition are shown in Table 3.  Figure 1 and web-only Figures A–D show in which cohorts and groups risk factors cluster, that is, ORs for observed versus expected number of children. From these analyses clustering was found for ≥4 risk factors in all cohorts except the 6-year-old cohort from Copenhagen (CoSCIS) (Figure 1). The number of children having ≥3 risk factors also showed clustering, but with a low OR (web-only Figure A).

Across cohorts, 6.2% of youth had 4+ risk factors and 15.7% had 3+ risk factors where a risk factor was defined as the upper quartile of risk (Table 3). OR for three risk factors was 1.09 (1.02–1.17) (online-only Figure 1B). The true number of children with clustering of CVD risk factors may be between 6.2% and 15.7%, and a conservative approach would be to define 6.2% with MetS. Cutoff in mean* z-*score for 6.2% was 0.85 and cutoff selecting 15.7% was 0.39.

### 3.1. Sensitivity Analyses (Table 4)

Analyses of composite MetS score were compared when sum of 4 skinfolds or BMI were used rather than waist circumference as the adiposity risk factor. The Pearson correlation between the mean* z*-scores including either BMI, waist circumference, or skinfold, respectively, was >0.97 for all associations. When the associations between CRF and dichotomized variables of the three mean* z*-scores were examined, greater magnitude of association was found when sum of skinfolds was used as the adiposity variable in the composite score, but similar magnitude was found for scores including waist circumference and BMI (Table 4). Sensitivity analysis was conducted to examine whether the magnitude of associations between CRF and the MetS score differed when HOMA was included in the score rather than fasting glucose, keeping other variables constant. The magnitude of association was greater when the MetS composite score included HOMA rather than fasting glucose (Table 4). Including APOA1, APOB, adiponectin, or CRP in the MetS score did not strengthen the association between CRF and MetS score even if they were associated with MetS score. However, leptin improved the composite MetS score substantially. OR for the least fit quartile increased from 18.3 (7.8–43.3) to 75.2 (18.2–309.8) when leptin was added to the MetS score. The strengthened association was apparent even if waist circumference was already part of the mean* z*-score.

Further, the importance of including CRF as a component of the MetS composite score was tested. To do this we needed an independent variable to analyze against MetS. Waist was therefore removed from the MetS score and used as independent variable. In this analysis the MetS consisted of systolic BP, TG, inverse HDL, and HOMA. Additional inclusion of CRF increased OR for the highest quartile of waist circumference from around 4 to 15, compared to the lowest quartile.

We also analyzed waist circumference and CRF, independently, as exposure variables against a MetS score including systolic BP, HOMA, TG, and inverse HDL. A higher OR was found for low CRF compared to high waist circumference. In the analysis of waist circumference, the 2nd and 3rd quartile of waist were not different from the lowest quartile and the upper quartile had an increased risk of 4.1 (95% CI 3.1–5.4) (Table 4). For CRF all three lower quartiles had higher risk than the most fit with an OR of 5.2 (3.8–7.2) for the least fit.

### 3.2. Testing a Noninvasive MetS Score

The noninvasive MetS composite score (mean of* z-*score for waist circumference/height and 1/CRF) was analyzed against the IDF definition of MetS using ROC analysis. A sensitivity and specificity of 0.85 and an area under the curve of 0.92 were found for a threshold point of 0.51 in* z*-score. When analyzed against the mean* z*-score of log waist, log TG, log HOMA, systolic BP, inverse HDL, and inverse fitness, a noninvasive mean* z*-score of 0.44 gave a sensitivity and specificity of 0.87 with an area under the curve of 0.94.

## 4. Discussion

Clustering of CVD risk factors could be viewed as a biological sign of poor metabolic health. The current analysis showed that more children had clustering of CVD risk factors than the number fulfilling IDF definition of MetS for children. More than 6.2% of children had clustering of 4 or more CVD risk factors in contrast to less than 1% according to the IDF definition. Changing the IDF diagnostic criteria to include more children would enable health professionals to start preventive initiatives much earlier and using a noninvasive screening tool may allow school teachers to start counseling health behavior in children at risk. If IDF definition only diagnoses about one-tenth of children with metabolic risk, a change in definition is necessary, because early prevention may be much more efficient than later treatment of manifest disease.

Previous definitions have used dichotomized risk factors to define MetS. The use of cutoff points in CVD risk factors could originate in previous use of guidelines to determine drug treatment, but drug treatment may not be the first choice in children. A continuous score includes full information of the health status from the risk factors. Continuous scores have recently been used by several groups [22, 23]. Some authors have postulated that* z*-scores are population specific [24]. However, this is only true if mean and SD from the specific population are used when variables are standardized. We have used common mean and SD of age and sex adjusted variables calculated from a great number of cohorts, which strengthen the generalizability of the score. We suggest changing existing dichotomized definitions to use continuous scores, because these use full information and can be easily calculated using Internet-based equations.

Sensitivity analyses showed that CRF was as strongly related to other CVD risk factors as waist circumference and logically should be included in MetS definition. Including leptin strengthened the association between MetS and an exposure substantially even if waist circumference was already included in the score. Inclusion of CRP, adiponectin, APOA1, and APOB did not strengthen the association further. A general practitioner is not expected to perform the CRF test, but all school physical education teachers can perform field tests of CRF with sufficient quality. A number of field tests are available, which allow whole school classes to be tested in one session [25, 26]. The IDF definition also uses fasting glucose as criteria for an adverse glucose homeostasis, which makes sense in adults where *β*-cell function has decreased. However, HOMA score provides a better estimate of impending or current impaired glucose metabolism in children. This view is supported by other studies [27]. Inclusion of other risk factors than the traditional IDF factors in calculation of mean* z*-score is easy and strengthens diagnostic criteria, which improve sensitivity and specificity. However, even if some risk factors are not measured, the mean* z*-score may still indicate a reasonable correct level of metabolic health.

A weakness of the IDF definition is the use of 90% percentile of waist circumference to define overweight [6]. This definition makes it difficult to compare populations, and it also hides secular trend in a population where a preventive strategy could be justified, because WC have gradually changed over time. Sensitivity analysis showed that fatness variables were interchangeable. This means that the use of continuous, age adjusted, and standardized variables in the definition of MetS makes it possible to compare risk levels between populations and over time even if studies have used different variables. However, we still recommend using waist circumference or BMI, because skinfold measurement is difficult to standardize. Variables included in the* z*-score should be constructed from a fixed mean and SD.

### 4.1. Strengths and Weaknesses

Assessment of the prognostic value of MetS in children for later CVD constitutes a weakness. The composite MetS score tracks into adulthood [28], but it is still uncertain how risk level in a child is related to later CVD. Further, we did not adjust for puberty or racial differences. However, in earlier analyses of all the European cohorts, adjustment for Tanner Stage did not change any estimates and racial differences were much smaller than cultural differences [29].

Inclusion of a large number of cohorts sampled from North-, East-, Mid-, and Southern Europe and North America enables comparison of consistency of observations between populations, which is a strength to this study. In these culturally diverse populations the clustering of risk factors occurred at a similar level of mean* z*-score. The approach is flexible in relation to which risk factors, covering a specific trait, are available.

Since Reaven described syndrome X almost 30 years ago, many new risk factors have been accepted as causal in the development of CVD and type 2 diabetes. When quartiles of CRF were analysed against a MetS outcome including systolic BP, TG, HOMA, and inverse of HDL, we found an even stronger association than was found for quartiles of waist circumference analysed against the same outcome. CRF may be the most important risk factor, which should have been included in definitions of MetS, but leptin also strengthened the association between the MetS variable and the independent variable. There is consensus that insulin resistance is a key factor in clustering of CVD risk factors and that muscle tissue is quantitatively the largest organ for glucose disposal. It has been shown that one-legged training can double glucose uptake in the trained leg compared to the untrained, which proves the importance of muscle tissue independent of abdominal obesity in the development of insulin resistance [30]. The methodology to diagnose MetS should include existing accepted risk factors but should allow for other emerging risk factors, which may prove important in future research. Inclusion of CRF and leptin improved the MetS score. Because we use the mean of standardized variables, scores are still comparable between studies. Calculations may seem cumbersome, but we have constructed a freely available software on the Internet to calculate scores, which makes it easy to use in general practise (http://www.video4coach.com/zscore/index.html).

In conclusion, the use of CVD risk factors as continuous variables can provide more information on MetS in children. Also, inclusion of other risk factors such as CRF and leptin and substituting glucose with HOMA score improved diagnostic criteria. This is important since most children do not have CVD but are in the developmental stages of MetS. The suggested approach solves many of the inherent problems in the previous definition formulated by IDF. Further, our noninvasive approach may be useful as a prescreening tool.

## Supplementary Material

Web appendix provide detailed information on absolute levels without age adjustment for CVD risk factors (Web Table 1) and also shows meta-analyses for observed versus expected number of children with ≥3 risk factors (Figure A), =3 risk factors (Figure B), =4 risk factors (Figure C), and =5 risk factors (Figure D).

## Figures and Tables

**Table 1:** Description of age adjusted risk factor levels for all cohorts. Adjustment for age enables comparison between cohorts.

Girls	Waist (cm)			Systolic BP (mmHg)			HDL (mmol/L)			Triglyceride (mmol/L)			HOMA		
	*N*	Mean	SD	*N*	Mean	SD	*N*	Mean	SD	*N*	Mean	SD	*N*	Mean	SD
Denmark 9 yr (EYHS)	547	18.9	2.6	547	104.4	8.2	547	1.47	0.31	547	0.88	0.38	547	2.00	.97
Denmark 15 yr (EYHS)	464	18.6	2.9	464	102.2	8.5	464	1.52	0.32	464	0.86	0.40	464	1.76	1.13
Portugal 9 yr (EYHS)	501	20.1	3.4	501	98.1	8.9	501	1.52	0.31	501	0.82	0.38	501	1.61	.87
Portugal 15 yr (EYHS)	378	19.1	3.2	378	96.3	8.2	378	1.56	0.31	378	0.63	0.35	378	1.29	1.02
Estonia 9 yr (EYHS)	303	18.2	2.3	303	103.8	8.9	303	1.40	0.29	303	0.83	0.28	303	1.71	.78
Estonia 15 yr (EYHS)	333	18.0	2.8	333	102.6	9.2	333	1.50	0.28	333	0.77	0.36	333	1.96	1.23
Norway 9 yr 2000 (EYHS)	196	18.7	2.4	196	105.5	8.3	196	1.45	0.30	196	0.97	0.30		·	·
Norway 15 yr 2000 (EYHS)	181	17.8	3.2	181	103.8	8.8	181	1.42	0.31	181	0.98	0.47		·	·
Denmark 6 yr (CoSCIS)	333	19.7	1.9	333	104.9	7.7	333	1.38	0.27	333	0.74	0.29	333	1.54	.45
Denmark 9 yr (CoSCIS)	338	19.0	2.6	338	106.4	8.9	338	1.54	0.37	338	0.63	0.24	338	1.64	.75
Denmark 13 yr (CoSCIS)	252	18.4	2.8	252	106.9	7.9	252	1.50	0.33	252	0.76	0.37	252	2.06	1.23
USA 6–10 yr (NHANES)	576	20.6	3.7	576	104.9	8.8	576	1.40	0.33	576	1.06	0.61		·	·
USA 10–14 yr (NHANES)	796	20.9	5.2	796	106.4	9.2	796	1.45	0.36	796	0.90	0.48	796	2.51	3.67
USA 14–18 yr (NHANES)	1046	21.0	5.8	1046	107.7	10.0	1046	1.38	0.30	1046	0.90	0.90	1046	1.91	3.37
Norway 9 yr 2005 (EYHS)	604	19.2	2.7	604	105.9	7.7	604	1.66	0.35	604	0.77	0.33	604	1.49	.71
Norway 15 yr 2005 (EYHS)	479	18.7	2.9	479	104.2	8.8	479	1.68	0.34	479	0.71	0.32	479	1.26	1.19
Switzerland 8 yr (KISS)	320	19.2	2.4	320	105.9	8.1	320	1.50	0.36	320	0.72	0.29	320	1.54	.66
Switzerland 12 yr (KISS)	345	18.5	2.9	345	107.5	8.8	345	1.62	0.37	345	0.64	0.32	345	1.46	.82

Boys	Waist (cm)			Systolic BP (mmHg)			HDL (mmol/L)			Triglyceride (mmol/L)			HOMA		
	*N*	Mean	SD	*N*	Mean	SD	*N*	Mean	SD	*N*	Mean	SD	*N*	Mean	SD

Denmark 9 yr (EYHS)	459	18.9	2.5	459	106.6	7.86	459	1.55	0.37	459	0.78	0.34	459	1.79	.94
Denmark 15 yr (EYHS)	391	18.4	2.7	391	109.4	10.99	391	1.41	0.28	391	0.79	0.48	391	1.83	1.64
Portugal 9 yr (EYHS)	538	20.1	3.3	538	99.0	8.59	538	1.55	0.32	538	0.74	0.35	538	1.33	.58
Portugal 15 yr (EYHS)	358	18.5	3.1	358	102.3	10.63	358	1.40	0.27	358	0.60	0.31	358	.91	.93
Estonia 9 yr (EYHS)	278	18.3	1.9	278	105.2	10.59	278	1.44	0.32	278	0.75	0.31	278	1.59	.68
Estonia 15 yr (EYHS)	260	17.9	2.6	260	109.8	11.97	260	1.39	0.27	260	0.65	0.32	260	1.80	1.58
Norway 9 yr 2000 (EYHS)	210	18.6	2.0	210	107.0	6.73	210	1.60	0.41	210	0.87	0.34	·	·	·
Norway 15 yr 2000 (EYHS)	167	17.9	2.4	167	111.0	9.95	167	1.31	0.31	167	1.02	0.58	·	·	·
Denmark 6 yr (CoSCIS)	373	19.6	1.7	373	105.9	7.79	373	1.36	0.26	373	0.71	0.22	373	1.55	.65
Denmark 9 yr (CoSCIS)	368	19.0	2.5	368	108.6	8.73	368	1.54	0.32	368	0.60	0.26	368	1.53	.76
Denmark 13 yr (CoSCIS)	259	18.1	2.6	259	109.4	9.10	259	1.46	0.28	259	0.74	0.38	259	1.80	1.20
USA 6–10 yr (NHANES)	620	20.8	3.7	620	105.3	9.36	620	1.33	0.30	620	1.04	0.49	·	·	·
USA 10–14 yr (NHANES)	775	22.2	5.7	775	104.9	9.77	775	1.40	0.33	775	1.02	0.54	775	2.92	2.18
USA 14–18 yr (NHANES)	988	21.6	6.2	988	101.5	9.49	988	1.55	0.34	988	0.81	0.52	988	2.00	3.42
Norway 9 yr 2005	702	18.9	2.5	702	106.8	7.71	702	1.73	0.40	702	0.69	0.32	702	1.41	.67
Norway 15 yr 2005	514	18.4	3.4	514	110.1	8.95	514	1.51	0.30	514	0.73	0.48	514	1.26	1.29
Switzerland 8 yr (KISS)	337	19.2	2.6	337	106.5	9.06	337	1.51	0.33	337	0.75	0.28	337	1.67	.75
Switzerland 12 yr (KISS)	390	18.5	2.6	390	105.4	8.69	390	1.61	0.35	390	0.68	0.30	390	1.58	.95

**Table 2:** Overall mean value and regression *β*-values with risk factors and age. *β*-values were used to calculate age adjusted risk factor levels.

Characteristic	Regression *β* for age					
	Girls			Boys		
	Mean	*β*	*P* <	Mean	*β*	*P* <
BMI (kg/m^2^)	19.6	0.704	0.001	19.6	0.699	0.001
Waist circumference (cm)	67.4	2.190	0.001	68.3	2.317	0.001
Ratio waist to hip circumference	77.8	−0.014	0.001	75.6	0.012	0.001
Sum of 4 skinfolds (mm)	42.9	2.453	0.001	33.5	0.743	0.001
Diastolic BP (mmHg)	61.5	0.386	0.001	62.0	0.442	0.001
Systolic BP (mmHg)	105.2	1.339	0.001	106.1	1.382	0.001
Total cholesterol (mmol/L)	4.29	−0.032	0.001	4.10	−0.070	0.001
HDL-cholesterol (mmol/L)	1.48	−0.019	0.001	1.48	−0.025	0.001
LDL-cholesterol (mmol/L)	2.40	−0.039	0.001	2.29	−0.044	0.001
Triglyceride (mmol/L)	0.80	0.025	0.001	.75	0.028	0.001
Glucose (mmol/L)	4.99	0.041	0.001	5.06	0.042	0.001
Insulin (pmol/L)	55.0	4.476	0.001	51.1	4.522	0.001
CRF (VO_2max_ [mL/min/kg])	42.5	−0.634	0.001	50.5	0.418	0.001
CRP (mg/dL)	.84	−0.015	NS	.74	0.006	NS
APOA1 (*μ*mol/L)	11.2	1.311	0.001	10.6	1.197	0.001
APOB (*μ*mol/L)	1.00	0.037	0.001	.94	0.032	0.001
Leptin (ng/mL)	.93	0.099	0.001	.37	−0.007	NS
Adiponectin (*μ*g/mL)	17.0	−0.396	0.001	15.2	−0.841	0.001
HOMA	12.6	1.111	0.001	11.8	1.129	0.001

**Table 3:** Distribution of children according to the number of risk factors^1^. The two next columns display the proportion of children having 4 or more risk factors and the cutoff point in the continuous *z*-score^2^, which select the same number of children at risk. The last column displays proportion having MetS according to IDF definition^3^.

Cohort	Number with the risk factors waist, sysBP, 1/HDL, TG, and HOMA						Total *N*	Proportion (%)	Mean *z*-score^2^	Proportion (%) using IDF definition^3^
	0 RF	1 RF	2 RF	3 RF	4 RF	5 RF		4 + 5 RF		
Denmark 9 yr (EYHS)	302	306	191	85	36	8	928	4.74	0.83	0.0
Denmark 15 yr (EYHS)	260	261	200	67	36	7	831	5.17	0.88	0.1
Portugal 9 yr (EYHS)	374	271	171	105	52	22	995	7.44	0.63	0.5
Portugal 15 yr (EYHS)	231	240	150	66	30	8	725	5.24	0.46	0.2
Estonia 9 yr (EYHS)	178	177	110	64	26	5	560	5.54	0.76	0.0
Estonia 15 yr (EYHS)	184	190	122	66	21	9	592	5.07	0.82	0.2
Denmark 6 yr (CoSCIS)	153	137	118	48	9	2	467	2.36	0.80	0.0
Denmark 9 yr (CoSCIS)	160	132	80	37	15	8	432	5.32	0.91	0.2
Denmark 13 yr (CoSCIS)	156	132	83	41	23	10	445	7.42	0.81	0.0
USA 10–14 yr (NHANES)	158	132	53	43	28	12	426	9.39	1.36	3.0
USA 14–18 yr (NHANES)	351	241	124	83	49	22	870	8.42	1.29	4.1
Norway 9 yr (EYHS 2005)	352	289	191	92	48	21	893	6.95	0.72	0.8
Norway 15 yr (EYHS 2005)	242	241	148	67	33	11	742	5.93	0.80	0.9
Switzerland 8 yr (KISS)	132	118	73	28	16	9	376	6.70	0.65	0.3
Switzerland 12 yr (KISS)	181	128	95	55	22	8	489	2.30	0.90	0.0

Total	3212	2793	1794	900	405	149	9871	6.20	0.85	0.9

^1^At risk factor is defined as the upper quartile.

^2^Mean *z*-score was calculated after ln-transformation of skewed risk factors.

^3^Proportion of children with MetS using IDF defined cutoff points as suggested by Zimmet et al. (2007) [6].

**Table 4:** Sensitivity analyses of changing one parameter in the MetS outcome with another covering the same trait. At the bottom CRF and waist circumference are compared against the same MetS outcome. For each comparison a cutoff point in *z*-score was chosen, which selected the same proportion of cases.

	Odds ratios (95% CI) for quartiles of fitness			
	1st quartile	2nd quartile	3rd quartile	4th quartile
MetS without fatness (TG + HOMA + BP − HDL)	5.2 (3.8–7.2)	2.0 (1.4–2.9)	1.8 (1.2–2.6)	1 (ref.)
MetS with waist	19.5 (10.8–35.1)	3.7 (2.0–7.1)	2.7 (1.4–5.3)	1 (ref.)
MetS with sum4skin	30.0 (15.3–58.6)	5.0 (2.4–10.3)	3.2 (1.5–6.8)	1 (ref.)
MetS with BMI	21.8 (12.2–39.3)	4.8 (2.5–9.0)	2.7 (1.4–5.2)	1 (ref.)
MetS with bioimpedance (%fat)^∗^	11.7 (5.2–26.6)	2.2 (0.8–5.6)	1.0 (0.3–3.0)	1 (ref.)
MetS with waist circumference^∗^	10.9 (4.2–28.4)	2.3 (0.8–6.9)	1.6 (0.5–5.2)	1 (ref.)

MetS with HOMA	19.5 (10.8–35.1)	3.7 (2.0–7.1)	2.7 (1.4–5.3)	1 (ref.)
MetS with glucose	13.0 (7.7–21.8)	2.3 (1.3–4.3)	1.2 (0.6–2.4)	1 (ref.)

MetS (TG + HOMA + sysBP + waist − HDL)	18.3 (7.8–43.3)	3.2 (1.2–8.6)	2.6 (1.0–7.1)	1 (ref.)
Adding leptin	75.2 (18.2–309.8)	7.6 (1.6–35.4)	5.2 (1.1–25.0)	1 (ref.)

MetS (TG + HOMA + sysBP + waist − HDL)	22.7 (10.8–47.7)	3.1 (1.3–7.5)	2.3 (0.9–5.6)	1 (ref.)
Adding APOA1	19.8 (9.8–40.1)	2.8 (1.2–6.4)	2.0 (0.8–4.8)	1 (ref.)
Adding APOB	24.3 (10.4–56.7)	4.5 (1.7–11.6)	2.5 (0.9–7.0)	1 (ref.)
Adding adiponectin	13.5 (6.5–27.9)	1.9 (0.8–4.9)	2.0 (0.8–4.7)	1 (ref.)
Adding CRP	12.7 (7.4–21.7)	2.8 (1.5–5.3)	1.8 (1.0–3.5)	1 (ref.)

MetS (TG + HOMA + BP − HDL) against CRF quartiles^#^	5.2 (3.8–7.2)	2.0 (1.4–2.9)	1.8 (1.2–2.6)	1 (ref.)
MetS (TG + HOMA + BP − HDL) against waist quartiles^#^	1 (ref.)	1.1 (0.8–1.6)	1.2 (0.9–1.7)	4.1 (3.1–5.4)
Adding CRF against waist quartiles	1 (ref.)	1.8 (1.1–3.0)	2.5 (1.5–4.1)	14.8 (9.6–22.9)

^#^These two analyses compare the strength between quartiles of fitness and quartiles of waist circumference against the same MetS outcome.

^∗^Data only available in CoSCIS, which is why estimates of quartiles of fitness in relation to MetS including waist circumference differ from analysis above.
