# Supplementary material for: A New Approach to Define and Diagnose Cardiometabolic Disorder in Children
Source: J Diabetes Res. 2015 Apr 6;2015:539835. doi: 10.1155/2015/539835 (PMC4402570; doi:10.1155/2015/539835)
Supplement: Supplementary file 1 — Web appendix provide detailed information on absolute levels without age adjustment for CVD risk factors (Web Table 1) and also shows meta-analyses for observed versus expected number of children with ≥3 risk factors (Figure A), =3 risk factors (Figure B), =4 risk factors (Figure C), and =5 risk factors (Figure D). [file 539835.f1.pdf]

Web-only Table 1. Descriptive of anthropometry and CVD risk factors

Girls	Age (year)	Height (cm)	Weight (kg)	BMI (kg/m²)	Waist (cm)	Sum of 4 skinfolds (mm)	Resting systolic BP (mmHg)	Resting diastolic BP (mmHg)
Cohort	Mean (SD)	Mean (SD)	Mean (SD)	Mean (SD)	Mean (SD)	Mean (SD)	Mean (SD)	Mean (SD)
Denmark 9 yr (EYHS)	9.7 (0.4)	139.1(6.4)	33.7(6.6)	17.3 (2.6)	59.6 6.6)	39.3 (18.5)	101.2 (8.2)	60.6 (5.9)
Denmark 15 yr (EYHS)	15.6 (0.4)	165.0 (6.7)	57.6 (9.2)	21.1 (3.0)	70.5 (7.0)	52.6 (18.3)	107.2 (8.5)	62.9 (6.0)
Portugal 9 yr (EYHS)	9.4 (0.5)	135.3 (6.8)	33.7 (8.4)	18.2 (3.4)	60.1 (7.4)	44.6 (19.7)	94.4 (9.0)	54.2 (5.9)
Portugal 15 yr (EYHS)	16.0 (0.7)	160.1 (5.9)	56.0 (9.0)	21.8 (3.1)	68.8 (7.4)	53.9 (17.0)	101.7 (8.1)	59.4 (6.2)
Estonia 9 yr (EYHS)	9.5 (0.5)	137.0 (6.8)	31.1 (6.3)	16.5 (2.3)	55.5 (5.5)	30.7 (13.5)	100.4 (9.0)	59.4 (6.9)
Estonia 15 yr (EYHS)	15.4 (0.5)	165.0 (6.1)	55.5 (8.9)	20.4 (2.9)	65.8 (5.6)	44.3 (15.0)	107.2 (9.2)	63.9 (6.6)
Norway 9 yr (EYHS 2000)	9.7 (0.3)	138.4 (6.3)	32.9 (6.0)	17.1 (2.4)	59.6 (6.0)	39.6 (17.7)	102.4 8.3)	63.1 (6.1)
Norway 15yr (EYHS 2000)	15.4(0.4)	166.9 (6.2)	56.0 (9.5)	20.1 (3.2)	70.2 (7.5)	52.9 (22.5)	108.4 (8.7)	65.5 (6.0)
Denmark 6 yr (CoSCIS)	6.7 (0.4)	122.0 (4.7)	24.0 (3.8)	16.1 (1.9)	55.7 (5.5)	29.2 (10.4)	97.6 (7.8)	57.6 (5.9)
Denmark 9 yr (CoSCIS)	9.5 (0.4)	138.7 (6.0)	33.4 (6.4)	17.3 (2.6)	61.9 (7.1)	37.3(17.5)	102.8 (8.8)	61.2 (6.2)
Denmark 13yr (CoSCIS)	13.3 (0.3)	162.4 (6.2)	51.1 (8.9)	19.3 (2.8)	66.1 (5.8)	39.0 (16.4)	108.7 (8.0)	61.9 (6.3)
USA 6-10 yr (NHANES)	8.0 (1.2)	129.1 (8.7)	30.2 (9.5)	17.8 (3.8)	61.5 (10.8)	-	100.8 (8.9)	53.2 (11.1)
USA 10-14 yr (NHANES)	12.2 (1.1)	154.1 (10.8)	50.9 (17.1)	21.1 (5.3)	74.4 (14.5)	-	106.7 (9.5)	55.3 (11.4)
USA 14-18 yr (NHANES)	16.2 (1.3)	173.0 (8.2)	72.0 (20.2)	23.9 (5.9)	82.6 (15.3)	-	113.4 (10.2)	59.6 (11.5)
Norway 9yr (EYHS 2005)	9.6 (0.4)	138.3 (6.8)	33.8 (7.1)	17.5 (2.7)	63.1 (7.7)	46.6 (24.7)	102.6 (7.7)	70.1 (6.9)
Norway 15yr (EYHS 2005)	15.5 (0.4)	165.9 (6.2)	58.3 (8.9)	21.2 (2.9)	73.4 (7.3)	60.2 (22.6)	109.0 (8.8)	72.9 (7.3)
Switzerland 8 yr (KISS)	8.3 (1.6)	131.4 (10.8)	29.3 (7.9)	16.7 (2.5)	58.0 (7.1)	28.4 (13.7)	100.9 (8.5)	60.2 (7.6)

Switzerland 12 yr (KISS)	12.06 (1.4)	152.5 (11.9)	43.8 (12.4)	18.5 (3.1)	66.2 (7.9)	34.2 (17.9)	107.6 (9.5)	64.3 (7.8)

Girls	HDL cholesterol (mmol/l)	Triglyceride (mmol/l)	Glucose (mmol/l)	Insulin (pmol/l)	VO_{2max} (ml/min/kg)	CRP (mg/l)	Leptin (umol/ml)	Adoponectin (umol/ml)	HOMA
Cohort	Mean (SD)	Mean (SD)	Mean (SD)	Mean (SD)	Mean (SD)	Mean (SD)	Mean (SD)	Mean (SD)	Mean (SD)
Denmark 9 yr (EYHS)	1.5 1(0.31)	0.83 (0.38)	5.03 (0.35)	50.2 (27.7)	44.3 (6.8)				1.6 (1.0)
Denmark 15 yr (EYHS)	1.45 (0.32)	0.94 (0.40)	5.00 (0.42)	71.8 (32.2)	41.5 (5.9)				2.3 (1.1)
Portugal 9 yr (EYHS)	1.57 (0.32)	0.75 (0.38)	5.12 (0.45)	36.0 (26.3)	-				1.2 (0.9)
Portugal 15 yr (EYHS)	1.49 (0.30)	0.72 (0.35)	5.15 (0.40)	57.5 (27.4)	33.2 (5.2)				1.9 (1.0)
Estonia 9 yr (EYHS)	1.44 (0.29)	0.77 (0.28)	4.90 (0.38)	41.6 (23.4)	44.6 (7.0)				1.3 (0.8)
Estonia 15 yr (EYHS)	1.43 (0.28)	0.85 (0.35)	5.00 (0.41)	77.2 (35.7)	37.4 (4.9)				2.5 (1.2)
Norway 9 yr (EYHS 2000)	1.50 (0.30)	0.92 (0.30)	-	-	41.8 (6.2)				
Norway 15yr (EYHS 2000)	1.36 (0.31)	1.06 (0.47)	-	-	39.2 (5.3)				
Denmark 6 yr (CoSCIS)	1.48 (0.26)	0.61 (0.29)	4.21 (0.39)	26.1 (15.7)	44.8 (5.4)				0.7 (0.5)
Denmark 9 yr (CoSCIS)	1.59 (0.37)	0.57 (0.24)	4.79 (0.49)	39.9 (20.1)	46.0 (6.2)	0.91 (1.47)			1.2 (0.7)
Denmark 13yr (CoSCIS)	1.48 (0.33)	0.79 (0.37)	4.80 (0.47)	72.5 (34.8)	44.9 (6.7)	0.65 (1.00)	1.07 (1.01)	12.84 (8.14)	2.3 (1.2)
USA 6-10 yr (NHANES)	1.48 (0.33)	0.97 (0.61)	-	-	-				
USA 10-14 yr (NHANES)	1.45 (0.36)	0.92 (0.48)	5.33 (1.38)	73.4 (61.3)	-				2.7 (3.7)
USA 14-18 yr (NHANES)	1.30 (0.30)	0.99 (0.91)	5.17 (0.50)	74.4 (77.9)	-				2.6 (3.4)
Norway 9yr (EYHS 2005)	1.70 (0.35)	0.72 (0.33)	5.10 (0.34)	33.7 (20.5)	45.6 (7.4)	1.00 (2.26)	0.64 (0.76)	18.71 (8.91)	1.1 (0.7)
Norway 15yr (EYHS 2005)	1.61 (0.34)	0.79 (0.32)	5.21 (0.37)	53.3 (30.7)	43.3 (6.9)	0.89 (2.23)	1.23 (0.88)	17.24 (8.50	1.8 (1.2)
Switzerland 8 yr (KISS)	1.56 (0.36)	0.64 (0.30)	4.64 (0.47)	33.6 (18.4)	-	0.74 (1.03)			1.0 (0.7)
Switzerland 12 yr (KISS)	1.62 (0.38)	0.65 (0.32)	4.73 (0.35)	48.2 (24.5)	-	0.66 (1.09)			1.5 (0.8)

Boys	Age (year)	Height (cm)	Weight (kg)	BMI (kg/m²)	Waist (cm)	Sum of 4 skinfolds (mm)	Resting systolic BP (mmHg)	Resting diastolic BP (mmHg)
Cohort	Mean (SD)	Mean (SD)	Mean (SD)	Mean (SD)	Mean (SD)	Mean (SD)	Mean (SD)	Mean (SD)
Denmark 9 yr (EYHS)	9.6 (0.4)	140.0 (6.6)	34.3 (6.6)	17.4 (2.4)	60.3 (6.3)	32.7 (16.1)	103.4 (7.8)	61.0 (6.2)
Denmark 15 yr (EYHS)	15.7 (0.4)	175.2 (7.2)	64.4 (10.4)	20.9 (2.7)	73.6 (7.3)	36.0 (19.1)	114.7 (10.9)	61.5 (6.6)
Portugal 9 yr (EYHS)	9.4 (0.5)	135.6 (6.4)	33.8 (8.3)	18.2 (3.3)	62.4 (7.4)	37.7 (22.5)	95.2 (8.7)	53.9 (6.5)
Portugal 15 yr (EYHS)	15.9 (0.6)	170.1 (6.9)	61.4 (10.9)	21.2 (3.1)	72.9 (7.1)	34.4 (17.0)	107.9 (10.7)	58.7 (6.4)
Estonia 9 yr (EYHS)	9.6 (0.5)	137.5 (6.4)	31.7 (5.4)	16.7 (1.9)	58.1 (4.3)	24.5 (9.5)	101.8 (10.6)	58.8 (7.8)
Estonia 15 yr (EYHS)	15.5 (0.6)	174.1 (7.8)	61.7 (10.2)	20.3 (2.6)	71.0 (5.4)	*28.3* (11.6)	114.9 (12.1)	62.7 (7.1)
Norway 9 yr (EYHS 2000)	9.7 (0.4)	139.9 (6.3)	33.4 (5.7)	17.0 (2.1)	61.1 (5.3)	32.4 (15.8)	103.6 (6.7)	62.3 (5.4)
Norway 15yr (EYHS 2000)	15.3 (0.3)	175.3 (7.3)	62.4 (9.6)	20.2 (2.4)	74.8 (7.9)	36.0 (18.0)	115.9 (10.0)	65.4 (6.1)
Denmark 6 yr (CoSCIS)	6.8 (0.4)	123.8 (5.0)	24.6 (3.6)	16.0 (1.7)	55.7 (5.0)	24.4 (9.0)	98.4 (7.9)	57.9 (5.7)
Denmark 9 yr (CoSCIS)	9.6 (0.4)	140.0 (5.7)	33.9 (6.1)	17.3 (2.3)	62.7 (6.9)	30.2 (14.7)	104.9 (8.7)	62.0 (6.0)
Denmark 13yr (CoSCIS)	13.4 (0.3)	164.2 (8.0)	51.7 (9.8)	19.1 (2.6)	68.5 (6.6)	32.2 (17.2)	111.5 (9.2)	61.5 (5.9)
USA 6-10 yr (NHANES)	8.0 (1.2)	129.2 (9.7)	30.6 (9.8)	18.0 (3.8)	62.4 (10.5)	.	100.9 (9.4)	53.4 (11.4)
USA 10-14 yr (NHANES)	12.1 (1.2)	153.4 (8.7)	53.1 (16.2)	22.3 (5.8)	76.7 (13.7)	.	105.1 (9.8)	57.3 (11.0)
USA 14-18 yr (NHANES)	16.2 (1.3)	160.9 (6.8)	63.7 (17.8)	24.5 (6.2)	82.4 (14.7)	.	107.6 (9.4)	61.3 (9.3)
Norway 9yr (EYHS 2005)	9.6 (0.4)	139.9 (6.3)	34.0 (6.5)	17.3 (2.5)	62.2 (7.3)	35.2 (20.1)	103.3(7.7)	70.3 (6.8)
Norway 15yr (EYHS 2005)	15.6 (0.4)	175.8 (7.2)	64.6 (12.1)	20.8 (3.4)	75.0 (8.9)	38.1 (22.4)	115.3(9.0)	74.3 (6.9)

Switzerland 8 yr (KISS)	8.2 (1.6)	130.5(11.2)	28.9 (8.6)	16.6 (2.6)	57.1 (7.0)	34.1 (17.4)	101.0 (9.2)	60.7 (7.6)
Switzerland 12 yr (KISS)	12.2 (1.9)	151.7 (9.8)	43.5 (10.3)	18.7 (2.8)	64.9 (7.0)	41.7 (17.1)	105.8 (9.2)	63.8 (8.1)

Boys	HDL-choleste (mmol/l)	Triglyceride (mmol/l)	Glucose (mmol/l)	Insulin (pmol/l)	VO_{2max} (ml/min/kg)	CRP (mg/l)	Leptin (umol/ml)	Adoponectin (umol/ml)	HOMA
Cohort	Mean (SD)	Mean (SD)	Mean (SD	Mean (SD)	Mean (SD)	Mean (SD)	Mean (SD)	Mean (SD)	Mean (SD)
Denmark 9 yr (EYHS)	1.61 (0.37)	0.73 (0.34)	5.13 (0.35)	42.9 (27.7)	49.4 (7.7)	.			1.4 (0.9)
Denmark 15 yr (EYHS)	1.31 (0.28)	0.89 (0.48)	5.24 (0.45)	70.5 (41.6)	52.1 (6.8)	.			2.4 (1.6)
Portugal 9 yr (EYHS)	1.62 (0.33)	0.68 (0.35)	5.19 (0.34)	26.6 (17.3)	.	.			0.9 (0.6)
Portugal 15 yr (EYHS)	1.29 (0.27)	0.70 (0.32)	5.33 (0.45)	44.7 (24.3)	48.6 (7.5)	.			1.5 (0.9)
Estonia 9 yr (EYHS)	1.51 (0.32)	0.69 (0.31)	5.11 (0.37)	36.6 (19.5)	50.3 (6.5)	.			1.2 (0.7)
Estonia 15 yr (EYHS)	1.30 (0.27)	0.74 (0.32)	5.21 (0.44)	69.8 (41.4)	49.5 (6.4)	.			2.4 (1.6)
Norway 9 yr (EYHS 2000)	1.66 (0.41)	0.81 (0.34)	.	.	47.8 (6.5)	.			
Norway 15yr (EYHS 2000)	1.22 (0.31)	1.11 (0.58)	.	.	49.8 (6.5)	.			
Denmark 6 yr (CoSCIS)	1.50 (0.26)	0.57 (0.22)	4.37 (0.44)	24.5 (21.1)	48.5 (5.9)	.			0.7 (0.7)
Denmark 9 yr (CoSCIS)	1.61 (0.32)	0.53 (0.26)	4.88 (0.52)	35.4 (23.5)	51.8 (6.9)	0.58 (0.73)			1.1 (0.7)
Denmark 13yr (CoSCIS)	1.42 (0.28)	0.77 (0.38)	4.96 (0.41)	62.5 (34.0)	52.6 (8.3)	0.67 (1.14)	0.36(0.59	12.33(7.69)	2.0 (1.2)
USA 6-10 yr (NHANES)	1.43 (0.30)	0.94 (0.50)	.	.	.	.			
USA 10-14 yr (NHANES)	1.39 (0.33)	1.03 (0.53)	5.13 (0.90)	92.8 (63.0)	.	.			3.1 (2.2)
USA 14-18 yr (NHANES)	1.44 (0.33)	0.92 (0.52)	4.97 (0.56)	81.2 (85.0)	.	.			2.7 (3.4)
Norway 9yr (EYHS 2005)	1.79 (0.40)	0.63 (0.32)	5.23 (0.37)	30.0 (19.2)	51.0 (8.3)	0.76 (1.86)	0.40(0.55)	17.89(8.85)	1.0 (0.7)

Norway 15yr (EYHS 2005)	1.42 (0.30)	0.82 (0.48)	5.41 (0.61)	52.6 (33.6)	55.5 (8.8)	0.86 (1.89)	0.35(0.52)	13.22(7.33)	1.8 (1.3)
Switzerland 8 yr (KISS)	1.61 (0.33)	0.65 (0.29)	4.52 (0.39)	36.6 (25.0)	.	0.86 (1.37)			1.1 (0.8)
Switzerland 12 yr (KISS)	1.60 (0.35)	0.69 (0.31)	4.64 (0.43)	53.9 (27.7)	.	0.57 (0.93)			1.6 (0.9)

Web-only figures from meta-analysis.

A) Odds ratios for each cohort of observed divided with expected number of children with 3 or more risk factors.

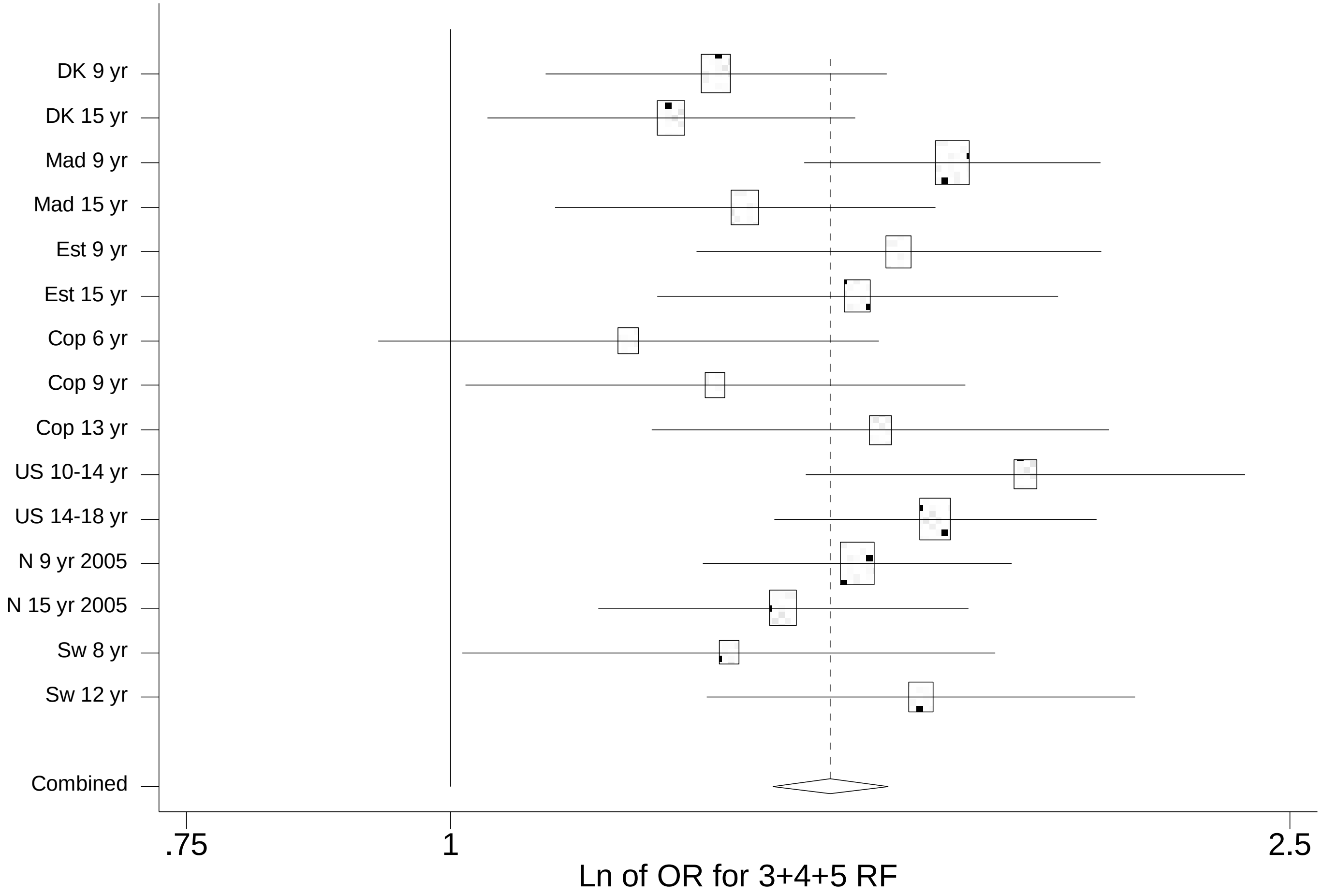

B) Odds ratios for each cohort of observed divided with expected number of children with 3 risk factors.

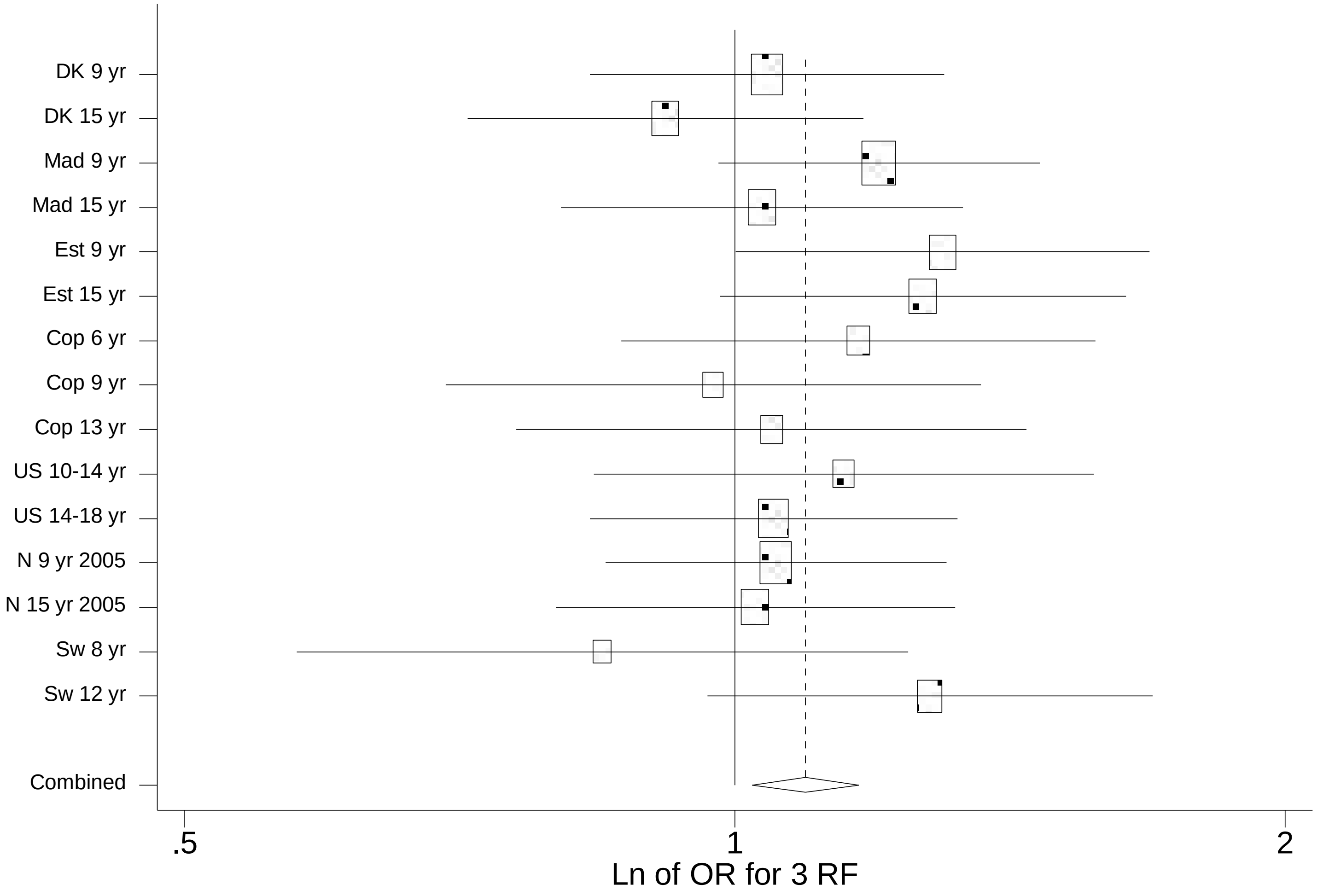

C) Odds ratios for each cohort of observed divided with expected number of children with 4 risk factors.

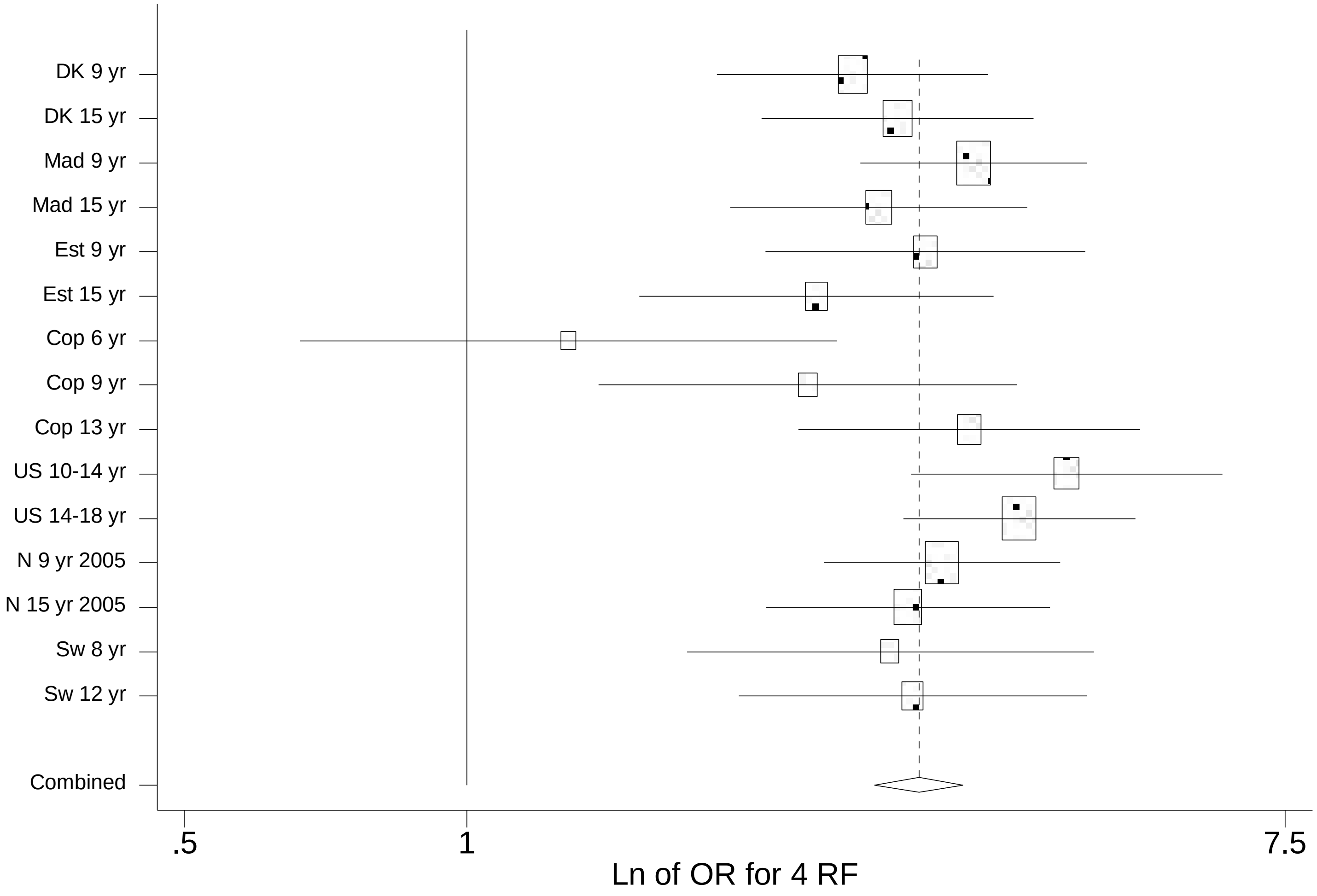

D) OR for each cohort of observed divided with expected number of children with 5 risk factors.

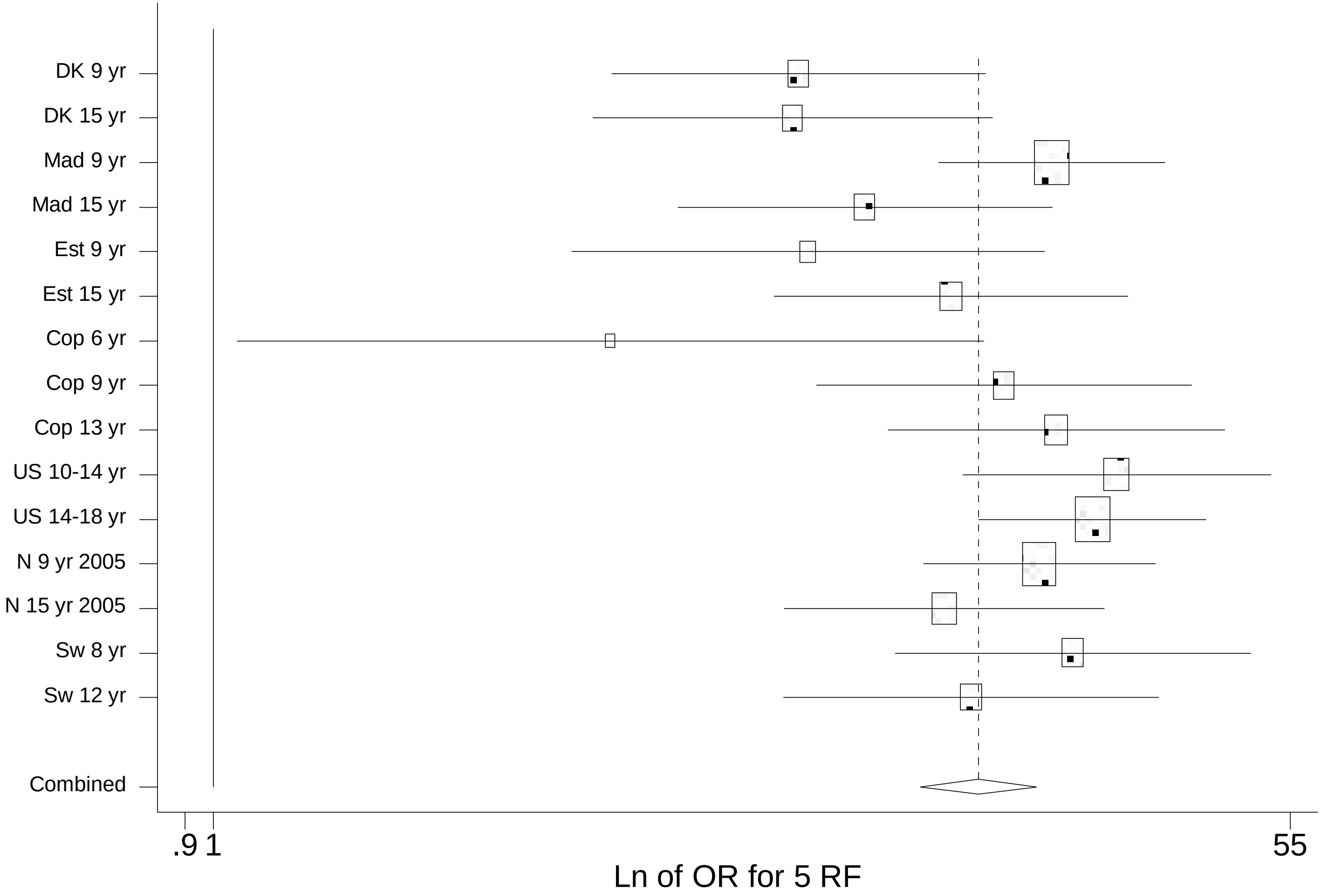

STROBE Statement—Checklist of items that should be included in reports of ***cohort studies***

	Item No	Recommendation
**Title and abstract**	1	(*a*) Indicate the study's design with a commonly used term in the title or the abstract See page 4 (abstract) and page 7 (methods)
		(*b*) Provide in the abstract an informative and balanced summary of what was done and what was found – page 4
**Introduction**		
Background/rationale	2	Explain the scientific background and rationale for the investigation being reported Page 5-7
Objectives	3	State specific objectives, including any prespecified hypotheses – page 6
**Methods**		
Study design	4	Present key elements of study design early in the paper – page 7
Setting	5	Describe the setting, locations, and relevant dates, including periods of recruitment, exposure, follow-up, and data collection – there are references to all included cohorts page 8
Participants	6	(*a*) Give the eligibility criteria, and the sources and methods of selection of participants. Describe methods of follow-up. Details from all 23 cohorts can be found in the published articles we have referred to on page 8.
		(*b*) For matched studies, give matching criteria and number of exposed and unexposed
Variables	7	Clearly define all outcomes, exposures, predictors, potential confounders, and effect modifiers. Give diagnostic criteria, if applicable. Page 8-9.
	8*	For each variable of interest, give sources of data and details of methods of assessment (measurement). Describe comparability of assessment methods if there is more than one group. This is done in the referenced studies page 8. Because we have pooled 23 cohorts, this information is not repeated here.
Bias	9	Describe any efforts to address potential sources of bias. We have controlled for conventional confounders. See page 9.
Study size	10	Explain how the study size was arrived at. We pooled available cohorts of children from different parts of Europe and North America.
Quantitative variables	11	Explain how quantitative variables were handled in the analyses. If applicable, describe which groupings were chosen and why. Normality was checked for continuous variables and skewed variables were log-transformed before they were standardized. See page 8-10.
Statistical methods	12	(*a*) Describe all statistical methods, including those used to control for confounding. Described in detail page 8-10.
		(*b*) Describe any methods used to examine subgroups and interactions. Estimates are described for each cohort separately, and a meta-analysis is also presented (Figure 1 and Table 3.
		(*c*) Explain how missing data were addressed. Representativity is not an important issue for the associations we analyse, and we did not impute data.
		(*d*) If applicable, explain how loss to follow-up was addressed. There is no follow-up.
		(*e*) Describe any sensitivity analyses. This is key findings in our study and is presented in Table 4.
**Results**		
Participants	13*	(a) Report numbers of individuals at each stage of study—eg numbers potentially eligible, examined for eligibility, confirmed eligible, included in the study, completing follow-up, and analysed. Not relevant.

		(b) Give reasons for non-participation at each stage. All observations are treated as cross-sectional.
		(c) Consider use of a flow diagram
Descriptive data	14*	(a) Give characteristics of study participants (eg demographic, clinical, social) and information on exposures and potential confounders. Provided in Table 1.
		(b) Indicate number of participants with missing data for each variable of interest. N is provided in all variables for all cohorts in Table 1. Participation rate is provided in the original articles from the cohorts referenced in the beginning of methods.
		(c) Summarise follow-up time (eg, average and total amount)
Outcome data	15*	Report numbers of outcome events or summary measures over time. Provided in Table 3.
Main results	16	(*a*) Give unadjusted estimates and, if applicable, confounder-adjusted estimates and their precision (eg, 95% confidence interval). Make clear which confounders were adjusted for and why they were included. All analyses are adjusted for sex and age. See Table 2.
		(*b*) Report category boundaries when continuous variables were categorized. Our main approach is to make composite scores of CVD risk factors using continuous variables (z-scores).
		(*c*) If relevant, consider translating estimates of relative risk into absolute risk for a meaningful time period
Other analyses	17	Report other analyses done—eg analyses of subgroups and interactions, and sensitivity analyses. See Table 4.
**Discussion**		
Key results	18	Summarise key results with reference to study objectives. Page 16.
Limitations	19	Discuss limitations of the study, taking into account sources of potential bias or imprecision. Discuss both direction and magnitude of any potential bias. See 'strenths and weaknesses' page 15-16.
Interpretation	20	Give a cautious overall interpretation of results considering objectives, limitations, multiplicity of analyses, results from similar studies, and other relevant evidence. Page 15-16.
Generalisability	21	Discuss the generalisability (external validity) of the study results. We have pooled data from 23 cohorts, which is a major strength in relation to generalizability.
**Other information**		
Funding	22	Give the source of funding and the role of the funders for the present study and, if applicable, for the original study on which the present article is based. The present study was not funded, but data collection in all included cohorts were funded. However, no funded source have been involved in this article.

*Give information separately for exposed and unexposed groups.

Note: An Explanation and Elaboration article discusses each checklist item and gives methodological background and published examples of transparent reporting. The STROBE checklist is best used in conjunction with this article (freely available on the Web sites of PLoS Medicine at http://www.plosmedicine.org/, Annals of Internal Medicine at http://www.annals.org/, and Epidemiology at http://www.epidem.com/). Information on the STROBE Initiative is available at http://www.strobe-statement.org.
